# Facilitating Change in Drinking Cognitions and Behaviors Among Three Immigrant Generations of Latinx Youth Through a School-Based Intervention: Findings From a Multi-Site Clinical Trial

**DOI:** 10.3389/fpsyt.2020.574487

**Published:** 2020-11-16

**Authors:** Guadalupe A. Bacio, Tracey A. Garcia, Kristen G. Anderson, Sandra A. Brown, Mark G. Myers

**Affiliations:** ^1^Departments of Psychological Science and Intercollegiate Chicana/o-Latina/o Studies, Pomona College, Claremont, CA, United States; ^2^Department of Psychology, Murray State University, Murray, KY, United States; ^3^Adolescent Health Research Program, Department of Psychology, Reed College, Portland, OR, United States; ^4^Departments of Psychology and Psychiatry, University of California, San Diego, La Jolla, CA, United States; ^5^Veterans Affairs San Diego Healthcare System and Department of Psychiatry, University of California, San Diego, La Jolla, CA, United States

**Keywords:** Latinx adolescents, alcohol expectancies, immigrant generation, adolescent alcohol use, alcohol cessation expectancies, alcohol use intervention, school-based intervention

## Abstract

Latinx youth experience disparities in the availability of and participation in evidence-based interventions to reduce hazardous alcohol use. The aim of this secondary data analysis was to examine whether Project Options, a brief, evidence-based alcohol use intervention was beneficial for Latinx participants. A total of 331 first-, second-, and third-generation immigrant Latina and Latino youth who participated in a multi-site, hybrid effectiveness/efficacy clinical trial of the intervention were selected for analyses. Mixed-effects growth models tested changes in drinking cognitions (i.e., perception of peer drinking, intention to drink next month, alcohol use and cessation expectancies) and behaviors (i.e., number of past-month drinking days, average number of drinks per occasion, and maximum number of drinks per occasion) across three time points (i.e., baseline, 4-weeks, and 12-weeks). Consistent with prior Project Options studies, participants with more drinking experience reported greater decreases in perception of peer drinking, intentions to drink next month, and all drinking behaviors than those with less experience. While no changes were observed in expectancies, first-generation participants endorsed lower positive use expectancies than second- and third-generation youth as well as more favorable cessation expectancies than third-generation teens. In concert with prior studies demonstrating the intervention's success in recruitment and retention of Latinx participants, results suggest that Project Options might be a promising school-based intervention for Latinx youth. This intervention has the potential to reach adolescents who might otherwise not participate in traditional programming and help decrease disparities in availability of evidence-based practices for Latinx youth.

## Introduction

Latinx^1^ youth are currently the largest ethnic group under the age of 18 in the United States (U.S.), and by 2060 they will account for 31.9% of all underage children, representing one of the fastest growing groups in the country (1). The rapid growth of Latinx youth only makes addressing the health disparities they face in the transition to adulthood even more pressing. For instance, while using alcohol and other substances is normative during adolescence [e.g., (2)], Latinx youth are at greater risk than their White counterparts to experience negative consequences (3, 4), less likely to have intervention services available (5), and less likely to complete treatment when enrolled (6). Thus, delivering evidence-based, culturally-responsive interventions for Latinx youth to address these disparities in service availability and utilization is a significant public health issue.

The disparities in alcohol use and related consequences among Latinx youth are exacerbated by a dearth of culturally-responsive, evidence-based interventions (EBIs) and compounded by low service utilization. Given the high need for intervention services and the many barriers to treatment faced by Latinx youth [e.g., cost, transportation, time; (7)], additional consideration has been given to programming accessibility or *reach*. Since attending school is compulsory for underage youth in the U.S., this setting offers an opportunity to reach Latinx youth that might otherwise not participate in traditional services. Furthermore, school-based interventions might be particularly apt to address key risk factors for underage drinking as schooling plays a significant role in socializing youth to peer norms (8, 9).

When and to what degree to adapt interventions for specific ethnocultural groups to increase EBIs' cultural responsiveness remains an ongoing discussion in intervention science. Several frameworks have been proposed to guide cultural adaptations that maintain fidelity to the EBI while improving ethnocultural fit [e.g., (10–13)]. While these frameworks provide valuable guidance in settings where a specific ethnocultural group can be easily targeted for intervention, the continuously changing ethnic composition of schools within districts and across geographical regions of the country complicates the implementation of culturally-responsive EBIs in a school setting.

Project Options is a brief, voluntary, cognitive-behavioral intervention, based on the premise of motivated, guided self-change that incorporates social cognitive components and developmental considerations important for adolescents. The model focuses on de-escalation of alcohol involvement and is grounded in a cognitive, social information processing approach (14). In this model, youth choose to reduce or stop drinking based on both distal and proximal cognitive and emotional factors. Targets for intervention include cognitive appraisal (e.g., perceived drinking norms; perceived prevalence of peer drinking behaviors) and evaluation processes (e.g., alcohol expectancies; beliefs about the effects of drinking alcohol), as well as improving skills that help youth manage deliberate and automatic-contextual temptations to drink (14–16). Project Options is adapted to the local context of each high school with the purpose of enhancing engagement across ethnic groups, genders, and levels of use but not specifically adapted to any particular ethnocultural group.

Efficacy studies demonstrated that Project Options attracts a diverse sample of youth (16, 17), that greater student participation in the program leads to higher levels of participant satisfaction (18), and that it facilitates youth change attempts in high frequency drinkers (14, 16, 17, 19). However, prior studies did not examine whether Project Options was effective for specific ethnocultural sub-groups.

Initial evaluations of a multi-site efficacy-effectiveness hybrid clinical trial of Project Options tested in three geographically and culturally different areas in the U.S. (i.e., Miami, FL, Minneapolis, MN, and Portland, OR) show that it is a promising EBI for Latinx youth. Specifically, a study of the intervention's voluntary recruitment and engagement strategies at each site demonstrated that participants more or less reflected the demographics of their corresponding school and that students who identified as African-American or Black where more likely to participate in the intervention than students of other ethnicities (20). Indeed, after attending one session, 79% of all participants were likely to voluntarily return to at least one more group. Similarly, an examination of the role of group ethnic diversity in therapeutic group processes among those in the motivational enhancement condition revealed that participants and interventionists in groups where the majority of participants (66% or higher) were African-American/Black or Latinx reported greater satisfaction and expressed more empathy than groups with non-Latinx white majorities (21). These findings suggest that the multi-site clinical trial showed promise for voluntarily attracting, retaining, and engaging Latinx youth in group content and positive therapeutic processes. However, it is currently unknown whether Project Options changed drinking cognitions (i.e., internalized thoughts and beliefs about alcohol use such as perceived prevalence of drinking among peers and the effects of drinking) and behaviors among Latinx participants.

Immigrant generation and gender are two important factors associated with alcohol use patterns and consequences among Latinx youth. Recent data indicate that 38% of the Latinx community are first-generation immigrants and only 34% are second-generation immigrant (U.S.-born of parents born in Latin America), while 28% are third-and-later generation immigrant [U.S.-born youth of Latin American ancestry whose parents are U.S.-born; (22)]. First-generation Latinx youth have been found to be less likely to start drinking in adolescence than their second-generation (23, 24) and third-and-later generation counterparts (23). Once first-generation youth begin drinking, they seem to drink at the same rate as second generation teens, but first- and second-generation youth report less problematic drinking than their third-and-later generation counterparts (23). These findings are consistent with the immigrant paradox [e.g., (25–27)], the pattern wherein first-generation immigrants seem to have more positive health outcomes than later generations despite the fact that immigrants experience multiple stressors before, during, and after immigrating to the U.S. (28).

Several hypotheses have been proposed to explain the immigrant paradox in adolescent drinking among Latinx youth. The acculturative stress hypothesis posits that the strain encountered by Latinx youth as they encounter challenges in adapting to mainstream U.S. culture may elicit a maladaptive stress response such as drinking alcohol [e.g., (29)]. Some have proposed that the loss of protective Latinx cultural practices across generations such as *familismo* [i.e., a sense of obligation to, deriving support from, and acting in reference to the family; (30)] and parental monitoring help explain the increased drinking behaviors among second- and later-generations compared to first-generation youth [e.g., (29, 31–33)]. Others have suggested that youth environments in the U.S. are risky and that increased exposure to risky behaviors and norms explains the immigrant paradox through factors such as association with deviant peers [e.g., (34–36)]. Studies that have tested multiple explanatory hypotheses simultaneously have shown that both the increased association with substance-using peers across generations (23) and perceptions of peer drinking prevalence (24) help explain the increased likelihood of drinking initiation among U.S.-born youth compared to their first-generation counterparts. In addition, the simultaneous generational decrease in family cohesion and increase in association with substance-using peers contribute to the exacerbation of problematic drinking observed among third- compared to second- and first-generation youth (23). Lastly, alcohol cognitions are linked to observed generational differences. For example, second-generation Latinx youth were found more likely to evaluate negative alcohol expectancies (i.e., beliefs about negative effects of alcohol; “If I drink, I will be more clumsy”) as “good”/desirable compared to first-generation youth. This difference in cognitions contributed to the finding that second-generation teens were more likely to initiate drinking in adolescence than were first-generation youth. Nevertheless, research to date does not identify a single explanation of the immigrant paradox in Latinx adolescent drinking. Rather, it seems that the mechanisms underlying this pattern are multidimensional and complex, often representing culture change processes at the adolescent, peer, family, and other ecodevelopmental levels (37). Less is known about whether generational differences are also observed in the context of intervention or treatment services aimed at reducing hazardous alcohol use.

Recent epidemiological studies have shown that Latinas are outpacing their Latino counterparts in some measures of alcohol use (38). For example, lifetime, current, and binge drinking is higher among Latinas compared to Latinos (38). This is troubling given that traditional Latinx households may hold stronger sanctions against alcohol use by girls than boys (39, 40). Few studies have examined how patterns of use by gender change across immigrant generational status (41, 42). However, there is some evidence to suggest that exposure to risky environments may have more influence on alcohol use behaviors for Latina than Latino teens. For example, Marsiglia et al. (43) showed that as Latinx students became more fluent in English, they were more likely to endorse pro-drug norms and, in turn, greater intentions for future use. These associations were observed among boys and girls, however, the mediating effect of pro-drug norms was stronger for Latinas than Latinos. Accordingly, some authors suggest that decreased parental monitoring and drinking restrictions for girls associated with longer time in the U.S., places Latinas at risk for negative drinking outcomes (44). At this time more research is needed to elucidate the mechanisms underlying the observed recent increases in alcohol use among Latinas compared to Latinos (38) and to ascertain whether the immigrant paradox might differ by gender. Differences by immigrant generation and gender on intervention provision and outcomes among Latinx youth have received even less attention.

While the literature has demonstrated the importance of drinking cognitions for understanding drinking behaviors, this area remains understudied among Latinx youth. Alcohol expectancies are cognitions that individuals develop regarding the probabilistic anticipatory effects of alcohol use that influence initiation and continued use of alcohol (e.g., people act like better friends after a few drinks of alcohol) (45, 46). The scant studies on Latinx youth replicate findings on general adolescent populations indicating that positive alcohol expectancies (expectation of positive outcomes from drinking) predict alcohol use (47–49). Even less is known about generational or gender differences in alcohol use expectancies among this group. Results from one study indicated that there might not be differences in positive or negative expectancies (expectations of poor outcomes from drinking) between first- and second-generation immigrants (24).

Among youth broadly, alcohol cessation expectancies, or expectancies about the consequences of stopping drinking or decreasing alcohol intake (50), are associated with lower rates of alcohol initiation among non-drinkers (51). Among drinkers, positive cessation expectancies predict less alcohol consumption and alcohol-related problems (50, 52). Research on cessation expectancies among Latinx adolescents is almost non-existent. One cross-cultural study found that Latinx students reported more peer-social and positive global cessation expectancies compared to the other ethnic groups (50).

Perceptions of peer alcohol use and intention to drink in the future are also important cognitions associated with concurrent and future alcohol use among adolescents. Peer perception of use is strongly associated with youth alcohol use over and above actual peer use (53, 54). Studies among Latinx youth indicate that second-generation adolescents endorse higher perception of peer use than their first-generation counterparts (24, 35). Importantly, these studies also demonstrated that this generational difference in perception of peer use mediated the relationship between immigrant generation and substance use. Further, intention to drink in the future captures motivation for actual behavioral change (14). While there are few studies that examine intentions among Latinx youth, drinking intentions have been found to prospectively predict alcohol use among primarily first-generation immigrant adolescents (39) and adolescents who identified as Mexican or Mexican American (55). More studies are needed to understand how these cognitions may differ by gender across immigrant generation.

### The Current Study

Evidence-based interventions aimed at reducing progression to hazardous alcohol use delivered in schools represent a promising avenue to reach underserved at-risk youth groups, including Latinx adolescents. While interventions adapted to the needs of specific ethnocultural groups are effective and, in some contexts, most appropriate (10, 13, 56), adapting school-based interventions for one ethnocultural youth group is not always indicated [e.g., (12)] or feasible given the diversity of student bodies. On the other hand, EBIs open to students regardless of ethnic background and experience with alcohol may be advantageous in reaching wide numbers of students. Project Options was developmentally tailored to the needs of adolescents, taking into consideration empirical findings regarding self-change processes and correlates of treatment efficacy. Preliminary studies suggest that Project Options successfully engaged Latinx youth across schools that differed in ethnic diversity and composition (20) and demonstrated positive group therapeutic processes for this ethnic group (21).

The purpose of this study was to conduct a secondary data analysis of the Project Options multi-site clinical hybrid efficacy/effectiveness trial to examine whether it is beneficial for Latinx youth. Consistent with the theoretical cognitive, social information processing model of the intervention, we examined changes across three time points (baseline, 4-weeks, and 12-weeks post-first session) in drinking cognitions (perception of peer drinking norms, alcohol use expectancies, alcohol cessation expectancies, and intention to drink) and drinking behaviors (past month drinking days, average number of drinks, and maximum number of drinks). Given findings from the initial Project Options trial (16), drinking experience was expected to moderate the changes in drinking cognitions and behaviors. Based on etiological studies of drinking patterns among Latinx youth, it was expected there would be differences at baseline in drinking cognitions and behaviors based on immigrant generation. First-generation participants were expected to evidence less positive alcohol expectancies, to view cessation expectancies more favorably, and perceive peer use to be less prevalent than second- and third-generation participants; differences between second- and third-generation youth were explored. Given recent trends in alcohol use by gender, it was expected that, at baseline, Latina participants would endorse riskier drinking cognitions and a higher number of drinking days and average number of drinks per drinking episode compared to their Latino counterparts.

Findings from this study will help determine whether Project Options, an EBI developmentally adapted to school settings, but not to specific ethnocultural groups, affects changes in drinking cognitions and behaviors among Latinx youth attending sociodemographically diverse schools. Further, this study contributes to the field's discussion of when to culturally adapt EBIs to enhance engagement and treatment effectiveness for Latinx youth.

## Methods

### Participants

A total of 460 Latinx students (39.42% of all participants) self-selected into Project Options, a voluntary, developmentally tailored, school-based, cognitive-behavioral intervention to reduce alcohol use (14, 16). The intervention was open to all students across eleven schools in Miami, FL, Minneapolis, MN, and Portland, OR between 2013 and 2016. Students were randomized to either a motivational enhancement (ME) delivery style or a didactic approach at a ratio of 3:1. For this study, a total of 331 Latinx participants for whom we collected immigrant generation data were selected for analysis (72% of Latinx sample). All participants completed a baseline assessment immediately prior to participating in the intervention, 80.36% completed a 4-week follow-up assessment (days: *M* = 29.43, *SD* = 4.78) and 66.77% participants completed a 12-week follow-up assessment (days: *M* = 86.76, *SD* = 5.89). Consistent with the overall demographics of each site, 80.05% of the analytic sample participated in Miami, 11.62% participated in Minneapolis, and 8.33% participated in Portland. Across sites, 65.65% of Latinx participants identified as girls. Approximately 29.31% were first-generation immigrant (i.e., immigrated to the U.S.), 50.45% were second-generation immigrant (i.e., U.S.-born of immigrant parents), and 20.24% were third generation (i.e., U.S.-born with one or two U.S.-born parents). Participants were 16.23 (*SD* = 1.44) years old on average. Table 1 illustrates participant demographics and key characteristics by immigrant generation.

**Table 1:** Demographic characteristics by immigrant generation.

	**Overall**	**First generation**	**Second generation**	**Third generation**
	***n* (%)/M(*SD*)**	***n* (%)/M(*SD*)**	***n* (%)/M(*SD*)**	***n* (%)/M(*SD*)**
**Total**	*N* = 331	97 (29.31%)	167 (50.45%)	67 (20.24%)
**Gender**				
Girls	216 (65.65%)	65 (30.09%)	109 (50.46%)	42 (19.44%)
Boys	113 (34.35%)	31 (27.43%)	57 (50.44%)	25 (22.12%)
**Age**	16.23 (1.44)	16.05 (1.54)	16.25 (1.39)	16.46 (1.41)
**Grade**				
9th Grade	75 (22.87%)	28 (37.33%)	33 (44.00%)	14 (18.67%)
10th Grade	61 (18.60%)	18 (29.51%)	33 (54.10%)	10 (16.39%)
11th Grade	71 (21.65%)	20 (28.17%)	35 (49.30%)	16 (22.54%)
12th Grade	121 (36.89%)	29 (23.97%)	66 (54.55%)	26 (21.49%)
**Country of ancestry**^e^				
Caribbean^a^	120 (36.36%)	54 (45.00%)	66 (55.00%)	0
Central America^b^	31 (9.39%)	12 (38.71%)	19 (61.29%)	0
North America^c^	62 (18.72%)	5 (8.06%)	14 (22.58%)	43 (69.35%)
South America^d^	46 (13.94%)	26 (56.52%)	20 (43.48%)	0
More than one country	71 (21.52%)	0	47 (66.20%)	24 (33.80%)
**Assessments**				
4-week follow-up	266 (80.36%)	88 (90.72%)	127 (76.05%)	51 (76.12%)
12-week follow-up	221 (66.76%)	66 (68.09%)	113 (67.66%)	42 (62.69%)
**Total number of sessions**	3.52 (1.84)	3.95 (1.62)	3.25 (1.90)	3.54(1.90)
**Lifetime drinking experience**				
0 Drinks	100 (30.40%)	36 (37.11%)	43 (25.90%)	21 (31.82%)
1–5 Drinks	118 (35.87%)	34 (35.05%)	63 (37.95%)	21 (31.82%)
6–20 Drinks	54 (16.41%)	11 (11.34%)	32 (19.28%)	11 (16.67%)
21+ Drinks	57 (17.33%)	16 (16.49%)	28 (16.87%)	13 (19.70%)

a *Countries reported: Cuba, Dominican Republic, Puerto Rico*.

b Countries reported: Costa Rica, Guatemala, El Salvador, Honduras, Nicaragua, Panama.

c Countries reported: Mexico, United States.

d Countries reported: Argentina, Bolivia, Chile, Colombia, Ecuador, Venezuela, Peru.

e *Country of ancestry reflects either the participants' or the participants' parents' country of birth*.

### Procedure

All high schools, respective school districts, and Institutional Review Boards approved procedures at each site. Information about Project Options was disseminated to students, parents, and teachers through flyers, posters, recurrent student newspaper ads, classroom and parent presentations, school websites, and newsletters. Advertisements were tailored to each school to appeal to students with different levels of alcohol experience and diverse backgrounds. Project Options was offered during lunch twice per week at each school by interventionists not affiliated with the schools to reduce impact on instructional time and maximize reach.

Based on prior adolescent self-change alcohol intervention research (16), Project Options protocol covered six topics: Perceived vs. Actual Alcohol Use Norms, Expectancy Effects/Balanced Placebo Studies, Managing Common & Uncommon Stress, Your Decisions/Consequences, Alternative Ways to Have Fun, and Communicating in Tough Situations. Participants could attend any session in no specific order regardless of drinking experience, up to six sessions. The specific language and style of materials were adapted to each site through focus groups. All interventionists were trained by Motivational Interviewing Network Trainers (MINT-certified) to deliver the intervention and supervised by a licensed clinical psychologist at each site.

The hybrid efficacy-effectiveness trial included two conditions with identical content, incentives, and length of session, but differed in *method* of content delivery: 1. A standard implementation of Project Options, including cognitive-behavioral skills building delivered in a motivational-enhancement, interactive, and collaborative style [ME; (16)], and 2. A didactic presentation of the same content wherein the cognitive-behavioral components were presented with limited interaction between students and where interventionists assumed a conventional expert/teacher role (57).

Students with parental consent self-selected into Project Options voluntarily. Participants determined the frequency with which they attended sessions independently and received free lunch (i.e., pizza) during session. Participants completed three assessments: immediately before their first session, ~4 weeks post-initial assessment, and 12 weeks post-initial assessment. All participants received a $5 gift card of their choice after the baseline assessment, a $10 gift card for their 4-week assessment, and a $15 gift card for their 12-week assessment.

### Measures

#### Demographics and Individual Characteristics at Baseline

Table 1 shows individual characteristics by immigrant generation.

##### Demographics

Participants endorsed whether or not they identified as Hispanic/Latino/a, and as a boy or girl (65.65%). Participants also reported their age (*M* = 16.23, *SD* = 1.44) and grade (9th = 22.87%, 10th = 18.60%, 11th = 21.65%, and 12th = 36.89%).

##### Immigrant generation

Participants wrote in the country in which they were born and the country in which their parents were born. All participants who reported having been born in a Latin American country were categorized as first-generation immigrants (29.31%). Those who were born in the U.S. and whose parents were born in Latin America were classified as second-generation immigrant (50.45%) while U.S.-born participants who reported that one or both of their parents were U.S.-born were categorized as third-generation immigrant (20.24%).

##### Lifetime drinking experience

Participants approximated the total number of times they drank alcohol over their lifetime by choosing: 0, 1–2, 3–5, 6–10, 11–20, 21–50, 51–100, and over 100. Due to the distribution of this variable, lifetime drinking categories were combined as follows: 0 (30.40%), 1–5 (35.87%), 6–20 (16.41%), and 21 or more (17.33%).

##### Number of sessions attended

The total number of sessions attended was calculated for each participant and ranged from 1 to 6 (*M* = 3.52, *SD* = 1.84).

#### Drinking Cognitions

Table 2 describes cognitive outcomes by immigrant generation at baseline assessment and Table 3 illustrates correlations among drinking cognitions and behaviors.

**Table 2:** Descriptive statistics at baseline by immigrant generation.

	**Overall**	**First generation**	**Second generation**	**Third generation**
	**%/M(SD)**	**%/M(SD)**	**%/M(SD)**	**%/M(SD)**
**Drinking cognitions**				
Perception of peer drinking	57.67%	48.66%	61.12%	62.04%
Alcohol expectancies	1.56 (0.97)	1.38 (1.05)	1.55 (0.96)	1.84 (0.85)
Cessation expectancies	2.85 (0.74)	3.01 (0.72)	2.80 (0.77)	2.75 (0.67)
Intention to drink next month	12.84%	7.45%	13.86%	17.86%
**Drinking outcomes**				
Number of drinking days	1.39 (3.93)	1.26 (3.93)	1.53 (3.31)	1.21 (2.46)
Average number of drinks per drinking episode	1.36 (3.88)	0.79 (1.56)	1.74 (5.14)	1.23 (2.12)
Maximum number of drinks per drinking episode	2.23 (5.16)	1.75 (4.63)	2.58 (5.87)	2.06 (3.78)

**Table 3:** Correlations among drinking cognitions and behaviors.

**Variable**	**1**	**2**	**3**	**4**	**5**	**6**	**7**	**8**	**9**	**10**	**11**	**12**	**13**	**14**	**15**	**16**	**17**	**18**	**19**	**20**
**DRINKING COGNITIONS**																				
**Perception of peer drinking**																				
1. Baseline	–																			
2.4-week follow-up	0.18^**^	–																		
3.12-week follow-up	0.16^**^	0.42^**^	–																	
**Alcohol expectancies**																				
4. Baseline	0.05	−0.05	−0.03	–																
5.4-week follow-up	0.03	−0.04	0.01	0.36^**^	–															
6.12-week follow-up	0.06	0.05	0.06	0.35^**^	0.37^**^	–														
**Cessation expectancies**																				
7. Baseline	0.01	0.07	0.02	−0.13^**^	−0.07	−0.14^*^	–													
8.4-week follow-up	0.06	0.11	0.08	−0.13^*^	−0.02	−0.02	0.40^**^	–												
9.12-week follow-up	−0.07	0.02	−0.13^*^	−0.10	−0.22^**^	−0.08	0.35^**^	0.46^**^	–											
**Intention to drink**																				
10. Baseline	0.01	−0.02	0.07	0.06	0.07	0.03	−0.13^**^	−0.11	−0.22^**^	–										
11.4-week follow-up	−0.02	−0.01	0.07	0.11^*^	0.15^*^	0.04	−0.09	−0.14^*^	−0.12	0.53^**^	–									
12.12-week follow-up	0.05	−0.06	−0.01	0.03	0.10	−0.01	−0.03	−0.05	−0.05	0.38^**^	0.39^**^	–								
**DRINKING OUTCOMES^a^**																				
**Number of drinking days**																				
13. Baseline	0.13^**^	0.08	<0.00	0.04	0.07	<0.00	−0.16^**^	−0.22^**^	−0.16^**^	0.37^**^	0.27^**^	0.21^**^	–							
14.4-week follow-up	0.09	0.10	0.14^*^	0.10	0.14^*^	0.09	−0.12^*^	−0.22^**^	−0.21^**^	0.44^**^	0.39^**^	0.29^**^	0.46^**^	–						
15.12-week follow-up	0.06	0.11	0.13^*^	0.12	0.12	0.05	−0.01	−0.24^**^	−0.15^*^	0.36^**^	0.34^**^	0.26^**^	0.27^**^	0.43^**^	–					
**Average number of drinks**																				
16. Baseline	0.07	0.11^*^	0.03	0.02	0.04	<0.00	−0.05	−0.20^**^	−0.11	0.18^**^	0.12^*^	0.19^**^	0.43^**^	0.25^**^	0.15^*^	–				
17.4-week follow-up	0.10	0.03	0.05	0.06	0.10	0.12	−0.03	−0.18^**^	−0.16^*^	0.27^**^	0.28^**^	0.32^**^	0.32^**^	0.59^**^	0.34^**^	0.32^**^	–			
18.12-week follow-up	0.06	0.08	0.10^*^	0.03	0.09	<0.00	−0.03	−0.22^**^	−0.20^**^	0.31^**^	0.28^**^	0.33^**^	0.35^**^	0.50^**^	0.61^**^	0.26^**^	0.43^**^	–		
**Maximum number of drinks**																				
19. Baseline	0.11^*^	0.10	0.09	0.03	0.02	0.01	−0.08	−0.16^**^	−0.11	0.25^**^	0.15^**^	0.29^**^	0.53^**^	0.29^**^	0.19^**^	0.82^**^	0.33^**^	0.26^**^	–	
20.4-week follow-up	0.11	0.05	0.07	0.08	0.15^**^	0.12	−0.07	−0.18^**^	−0.18^**^	0.31^**^	0.29^**^	0.33^**^	0.42^**^	0.61^**^	0.34^**^	0.41^**^	0.91^**^	0.43^**^	0.44^**^	–
21.12-week follow-up	0.05	0.09	0.13^*^	0.11	0.12	<0.00	−0.01	−0.22^**^	−0.20^**^	0.36^**^	0.41^**^	0.36^**^	0.36^**^	0.52^**^	0.76^**^	0.22^**^	0.47^**^	0.88^**^	0.25^**^	0.47^**^

* *p < 0.05*,

** *p < 0.01*.

a *Drinking outcomes were measured 30 days prior to assessment point*.

##### Perceived peer norms of alcohol use

Participants reported the percent of students in their grade they thought drank alcohol in the past month on a range from 0 to 100 (58).

##### Alcohol use expectancies

Anticipated expectancies of drinking alcohol (i.e., beliefs about the effects of alcohol) were assessed with two items (59): “Parties are not as much fun if people are drinking alcohol” and “People act like better friends after a few drinks of alcohol.” Participants rated each statement on a 5-point scale ranging from 1 “Strongly Disagree” to 5 “Strongly Agree.” Answers to these statements were averaged to calculate an alcohol expectancies score.

##### Alcohol cessation expectancies

Anticipated effects of cutting down or quitting alcohol use were assessed with two items (60): “The future would be” and “Fitting in with others would be” if someone their age would cut down or stop drinking alcohol. Participants rated each statement on 5-point scale ranging from “A lot worse” to “A lot better.” A cessation expectancies score was calculated by averaging these 2 ratings.

##### Intention to drink

Participants reported their intention to drink next month on a 5-point scale ranging from 1 (Definitely Not Drink) to 5 (Definitely Will Drink). Due to the distribution of responses, those who endorsed that they would definitely drink were compared to all other categories combined.

#### Drinking Behaviors

Table 2 illustrates descriptive statistics for drinking behaviors by immigrant generation at baseline assessment and Table 3 shows correlations among drinking cognitions and behaviors.

##### Drinking behaviors in the past month

Items were adapted from the Monitoring the Future Survey (61) to assess current alcohol involvement at each assessment point (i.e., during the 30 days prior to assessment). Participants reported the number of days they drank at least one drink of alcohol, the average number of drinks they had on the days they drank, and the maximum number of drinks they had on any drinking day.

### Analytic Plan

Mixed-effects growth models were used for all analyses to account for the nesting of repeated observations within participants (i.e., baseline assessment, 4-week follow up, and 12-week follow up). Analyses were conducted as intent-to-treat (i.e., all participants were included regardless of whether they had completed any follow up assessment). All participants regardless of drinking experience were included in all models. We accounted for the overdispersion of zeroes in drinking outcomes by using negative binomial mixed growth models. All likelihood ratio χ^2^ tests comparing negative binomial models to Poisson models were significant, indicating that the negative binomial models provided better estimates. We used mixed growth models to test changes in alcohol use and cessation expectancies as well as peer perception of alcohol use and logistic mixed growth models to test intention to drink next month. Based on prior findings of the intervention's effectiveness for risky drinkers (16), we examined whether lifetime drinking experience moderated intervention effects by testing an interaction between lifetime drinking experience by follow-up assessment.

Randomization of treatment condition (i.e., ME vs. didactic) was completed at the school level; schools served as their own control (i.e., treatment condition was switched within schools after a washout period). Since Project Options was not adapted to any ethnocultural group and open to everyone regardless of drinking experience, some sites had uneven distributions of Latinx participants at each lifetime drinking level. For these reasons, we were unable to nest models at the site or school level. To account for nesting by site, we included site as a covariate in each model. Time was modeled as the number of days since baseline. All models included participant gender, immigrant generation, lifetime drinking experience, total number of sessions attended, delivery style (ME vs. didactic), site, and time since baseline, and these were modeled as Level 1 fixed effects. Observations nested within participants were modeled as Level 2 fixed effects. Modified sandwich variance estimators were used in all models to account for non-normality and non-independence of observations by participants (62–64). Analyses were conducted using Stata 15.

## Results

### Drinking Cognitions

#### Perceived Prevalence of Peer Drinking

The linear growth model testing changes in perceived percentage of peer drinking was significant [Wald χ^2^(14) = 170.30, *p* < 0.001]. The interaction between lifetime drinking experience and follow-up assessments was significant [χ^2^(3) = 18.84, *p* < 0.001]. Compared to non-drinkers at baseline, those in the 1–5 (*b* = −0.195, *p* < 0.001), 6–20 (*b* = −0.191, *p* = 0.002), and 21 or more (*b* = −0.150, *p* = 0.007) categories reported greater decreases in perceived prevalence of peer drinking. In addition, the main effects of total number of sessions attended (*b* = −1.46, *p* = 0.030) and gender (*b* = −5.68, *p* = 0.027) were significant. That is, attending more sessions decreased the perceived prevalence of peer drinking. Similarly, boys endorsed lower levels of perceived prevalence of peer drinking, on average, than girls. The main effects of condition, site, and immigrant generation were not significant.

#### Alcohol Expectancies

Though the model testing the interaction between baseline lifetime drinking experience and assessments in predicting alcohol expectancies was significant, the interaction was not; therefore, the main effect model is presented [Wald χ^2^(11) = 32.01, *p* = 0.001]. Lifetime drinking experience [χ^2^(3) = 10.02, *p* = 0.018], immigrant generation [χ^2^(2) = 7.96, *p* = 0.019], and site [χ^2^(2) = 7.62, *p* = 0.022] independently predicted alcohol expectancies. Specifically, those who reported 6–20 (*b* = 0.378, *p* = 0.003) and 21 or more (*b* = 0.028, *p* = 0.029) drinks endorsed more positive alcohol expectancies compared to non-drinkers; no significant differences were found in any other comparisons. Participants in Minneapolis reported more positive alcohol expectancies compared to those in Miami (*b* = 0.447, *p* = 0.006), and there were no site differences between Portland and the other two sites. First-generation participants reported lower positive alcohol expectancies than their second- (*b* = 0.25, *p* = 0.014) and third-generation counterparts (*b* = 0.313, *p* = 0.012), while there were no differences between second- and third-generation participants. Condition, number of attended sessions, assessment time point, and gender did not have main effects on alcohol expectancies.

#### Cessation Expectancies

The model testing the interaction of lifetime drinking experience and assessment time point in predicting cessation expectancies was significant, but the interaction was not, therefore, the main effects model is presented [Wald χ^2^(11) = 52.55, *p* < 0.001]. Lifetime drinking experience [χ^2^(3) = 17.36, *p* = 0.001], condition (*b* = 0.313, *p* = 0.012), and generation (χ^2^(2) = 10.27, *p* = 0.006) had significant main effects on cessation expectancies. Those who reported 21 or more drinks at baseline endorsed more negative cessation expectancies compared to non-drinkers (*b* = −0.426, *p* < 0.001). There were no differences between those in the 1–5 and 6–20 categories compared to non-drinkers, respectively. Similarly, those randomized to the ME condition reported worse cessation expectancies (*b* = −0.252, *p* = 0.002). While third generation immigrants endorsed worse cessation expectancies compared to their first generation counterparts (*b* = −0.301, *p* = 0.001), there were no differences between third- and second- as well as first- and second-generation immigrants. Site, assessment time point, total number of sessions, and gender had no main effects on cessation expectancies.

#### Intention to Drink

The model examining intentions to drink next month was statistically significant [Wald χ^2^(14) = 45.44, *p* < 0.001]. The interaction between lifetime drinking experience and assessment time point was significant [χ^2^(3) = 13.96, *p* = 0.003]. Compared to non-drinkers at baseline, intention changes between those in the 1–5 lifetime drinks category (*b* = 0.962, *p* = 0.003) and those in the 21 or more lifetime drinks category (*b* = 0.968, *p* = 0.015) were significantly different. Similarly, the rate of change in intentions to drink next month was significantly different between those in the 6–20 baseline lifetime drinks category and those in the 1–5 drinks category (*b* = 1.03, *p* = 0.002) and between those in the 6–10 category and those who reported 21 or more drinks at baseline (*b* = 0.969, *p* = 0.013). The main effects of condition, total number of attended sessions, site, gender, and immigrant generation on intention to drink next month were not significant.

### Drinking Behaviors in the Past Month

#### Number of Drinking Days

The negative binomial growth model testing changes in number of drinking days in the past month was statistically significant [Wald χ^2^(14) = 223.52, *p* < 0.001]. The interaction between lifetime drinking experience and assessment time point was significant [χ^2^(3) = 20.97, *p* < 0.001]. There were significant differences in the rate of change in number of drinking days between those in the 1–5 (*IRR* = 0.979, *p* = 0.013), 6–20 (*IRR* = 0.973, *p* = 0.001), and 21 or more drinks (*IRR* = 0.968, *p* < 0.001) categories compared to non-drinkers. Similarly, the rates of change between those in the 21 or more drinks and those in the 1–5 category (*IRR* = 0.989, *p* = 0.025) were significantly different. The main effects of condition, site, total number of attended sessions, gender, and generation were not significant.

#### Average Number of Drinks per Drinking Episode

The negative binomial growth model that examined changes in average number of drinks per drinking occasion was significant [χ^2^(14) = 146.17, *p* < 0.001]. The interaction between drinking experience and assessment time point was significant [χ^2^(3) = 17.62, *p* = 0.001]. Compared to non-drinkers, the rates of change in average number of drinks per drinking occasion were significantly different by those who reported 1–5 (*IRR* = 0.975, *p* = 0.008), 6–20 (*IRR* = 0.970, *p* = 0.001), and 21 or more (*b* = 0.969, *p* < 0.001) drinks at baseline. Condition had a main effect on average number of drinks such that those randomized to the ME approach reported a rate of average number of drinks 1.757 times greater than those in the didactive condition (*z* = 2.17, *p* = 0.030). Site, total number of attended sessions, gender, and generation did not have significant main effects of average number of drinks per occasion reported by participants.

#### Maximum Number of Drinks per Occasion

The negative binomial growth model that tested changes in maximum number of drinks per occasion was significant [χ^2^(14) = 212.52, *p* < 0.001]. The interaction between drinking life experience and assessment time point was significant [χ^2^(3) = 17.55, *p* = 0.001]. There were significant differences in the rates of change between those who reported 1–5 (*IRR* = 0.967, *p* = 0.001), 6–20 (*IRR* = 0.965, *p* < 0.001), and 21 or more (*IRR* = 0.963, *p* < 0.001) drinks at baseline compared to non-drinkers. Condition had a marginally significant main effect on maximum number of drinks such that those randomized to the ME condition reported a rate of 1.68 times greater than those in the didactic condition (*z* = 1.93, *p* = 0.054). Total number of attended sessions, site, gender, and immigrant generation did not have main effects on the maximum number of drinks per drinking occasions in the past month reported by participants.

## Discussion

While strides have been made in the past few decades to improve the cultural responsiveness and reach of adolescent drinking interventions, Latinx youth continue to experience disparities in the availability of, participation in, and completion of evidence-based interventions. The purpose of this secondary data analysis was to examine whether Project Options, an alcohol use EBI adapted to the school setting but not to any specific ethnocultural group, was beneficial for Latinx participants in a real-world setting. Project Options is a motivationally enhanced, brief, cognitive-behavioral intervention that was designed to be voluntary, developed for adolescents, and open to students regardless of drinking experience. Using data from a hybrid effectiveness-efficacy multi-site clinical trial of the EBI, we tested changes across three assessment time points (i.e., baseline, 4-weeks, and 12-weeks) in drinking cognitions and behaviors by lifetime drinking experience among 331 first-, second-, and third-generation immigrant Latinx participants. Consistent with our hypotheses, Latinx participants with more lifetime drinking experience evidenced changes in drinking cognitions (i.e., perceptions of peer drinking norms, intention to drink next month) and behaviors (i.e., past month drinking days, average number of drinks per occasion, and maximum number of drinks per occasion) regardless of immigrant generation or gender.

Participant changes in drinking cognitions and behaviors did not differ by the method in which the information was provided (e.g., treatment condition: ME vs. didactic). This finding differs from the comparison of Project Options (ME condition) to Assessment Only condition in prior studies (16, 19). However, no differences comparing two active treatment conditions (ME vs. didactic) emerged in this study. Though we cannot fully attribute observed changes to participation in Project Options because the motivational enhancement delivery approach is posited to be an elemental part of the EBI, prior evaluations of intervention efficacy and positive findings from this multisite efficacy-effectiveness hybrid trial provide some evidence that participation in Project Options leads to the changes in drinking cognitions and behaviors reviewed below. The lack of condition effects may be due to several reasons. First, it may reflect the challenges inherent in discerning differential effects in relation to an active control condition that delivered identical content and differed only in *style* of delivery. Second, it is possible that the effect of intervention style was not sufficient to enhance outcomes over and above the effect of intervention content, which was based on theoretical and empirical evidence and was developmentally tailored to adolescents in school contexts. In fact, both conditions utilized in the multisite Project Options trial incorporated materials for which prior studies provided initial evidence of utility for changing drinking behavior when compared with a no-treatment control condition (16, 19). A third possibility is that we had limited statistical power to detect a differential effect of condition style. Though interventions based on ME principles are effective in changing substance use behaviors among adolescents, the effect size tends to be small on average (65). In addition, only a relatively small proportion of those self-selecting into this trial had substantial drinking experience. As such, relatively few participants were likely to feel ambivalent or concerned about their drinking and thus be amenable to motivational enhancement. A key precept of motivational interventions is the evocation and reinforcement of change talk (i.e., desire to change) which presupposes that participants have internalized concerns about their drinking behaviors (66). As such, the possibility of eliciting change talk could only be expected for a subset of intervention participants. This combination of factors may have decreased our ability to detect a difference between the two group-based treatment conditions. Fourth, condition randomization did not account for participant drinking profiles. For example, participants randomized to the ME condition reported a higher average number of drinks per occasion and worse cessation expectancies than those in the didactic style condition, thereby potentially concealing condition differences. Fifth, there is growing recognition that therapeutic *common factors*, including therapeutic alliance, as well as cultural sensitivity and content, can complicate distinguishing active treatment effects from a placebo condition (67). Though interventionists were asked to take a more conventional teacher role when delivering the didactic condition style, they still had to establish a therapeutic alliance with participants, which other studies have positively linked to treatment outcomes (68, 69). Lastly, the intervention content in both delivery styles was contextualized to fit cultural features of the schools (e.g., language/lingo) in which they were administered which could have obscured condition differences. Although there were no differential condition style effects, given prior evidence for efficacy from the initial EBI trials when compared with a no-treatment control condition (14, 16, 19) and the observed changes across time evidenced by the multi-site trial participants, we interpret the present multi-community findings to provide tentative support for the value of Project Options content as a school-based intervention for addressing alcohol use by Latinx youth.

### Changing Drinking Cognitions

This secondary data analysis of Project Options evaluated changes in four drinking cognitions: intention to drink next month, perception of peer drinking prevalence (i.e., beliefs about the percentage of peers who drink), positive alcohol expectancies (i.e., beliefs that alcohol has positive effects), and cessation expectancies (i.e., beliefs of whether things are better or worse if people reduce or quit drinking alcohol). As expected, Latinx youth with more drinking experience reduced their intention to drink and increased their accuracy of perception of peer drinking more so than those with less or no experience. This suggests that more experienced participants were more likely to be motivated to decrease or quit drinking alcohol. On the other hand, this could also be the result of the fact that those with more drinking experience have more room, or range in use, to change than those with less drinking experience. These results are consistent with our findings on drinking behaviors discussed below. In addition, attending more sessions was associated with greater reductions in perceived peer drinking prevalence. Further, Latino participants on average endorsed lower prevalence of peer drinking than their Latina counterparts. These findings are consistent with other studies (70) and prior EBI trials (19). Perception of peer behaviors is a strong predictor of initiation of use as well as intensity of substance use among Latinx youth across gender (8) and changing this cognition is related not only to concurrent drinking (71) but also to future drinking patterns (39, 72) regardless of drinking experience. The higher endorsement of peer drinking norms among Latinas across immigrant generations in this study is troubling given that it contradicts traditional gender norms among the Latinx community (73). This gender difference may be due to the greater number of Latina than Latino participants across generations in this sample. Further, this gender discrepancy might also reflect the observed increases in drinking behaviors reported by Latinas compared to Latinos at the national level (38). Finally, this disparity might reflect a potential differential impact of exposure to U.S. risky drinking norms. For instance, time in the U.S. across first- and later generations and increased English fluency has been associated with positive substance use norms more strongly for Latinas than Latinos (43, 44), signaling an erosion of protective traditional Latina drinking norms. This suggests that more studies are needed to understand the mechanisms that seem to be placing Latina youth at greater risk for potentially problematic drinking cognitions. Further, future intervention efforts are sorely needed to address this emerging gender disparity among Latinx youth.

Whereas, no changes were observed in expectancies for alcohol use and cessation across time, there were significant differences by immigrant generation in these two cognitions. Specifically, first-generation immigrants endorsed less favorable positive alcohol expectancies (i.e., fewer expectations of positive effects from drinking alcohol) than their second- and third-generation counterparts while first-generation participants also perceived reducing or quitting drinking more favorably (i.e., greater expectations that decreasing/quitting alcohol made things better) than their third-generation counterparts. While there are very few studies examining generational differences in alcohol outcome expectancies and none to our knowledge that have evaluated cessation expectancies *within* Latinx youth, these findings are in line with other studies (24). In addition, these differences map on to differences in drinking behaviors by immigrant generation among youth (23, 24, 35) and adults [e.g., (25)] that reflect the immigrant paradox in drinking patterns. While we could not test explanations of this paradox directly, these findings are consistent with the hypothesis that exposure to risky behavioral norms and behaviors inherent in youth environments in the U.S. helps explain why first-generation youth report less risky drinking behaviors than later generations (23, 24, 35). For example, studies have shown that immigrant youth have peer networks that are less prone to drink because they are more likely to have friends who are also immigrants due to placements based on English proficiency at school and a preference for Spanish speaking peers (74–76). In addition to being more likely to have lived in the U.S. for less years than Latinx youth of later generations, these first-generation teen peer networks seem to protect them from risky drinking norms and behaviors. Although no generational differences in drinking behaviors were observed in this sample, it is possible that this variance in beliefs about drinking and reducing/quitting drinking may portend differences in future drinking behaviors consistent with the immigrant paradox.

### Changing Drinking Behaviors

As expected, participants with more baseline lifetime drinking experience reduced the number of days they drank, as well as the average, and maximum number of drinks they consumed per drinking occasion across time. Neither gender nor immigrant generation were associated with these changes, suggesting that participating in Project Options might have been beneficial for Latinx participants regardless of participants' immigrant generation and gender. These findings are in line with prior efficacy trials of the EBI. For example, Schulte et al. (19) found that regardless of intervention attendance, the greatest reductions in drinking behaviors were observed among the heaviest drinkers. In sum, while Project Options was open to all Latinx participants regardless of drinking experience, it seems to be more beneficial for heavier drinkers over and above immigrant generation and gender.

In concert, findings from this multi-site hybrid efficacy/effectiveness trial suggest that Project Options content might have changed drinking cognitions and behaviors among Latinx participants. Prior studies of this multi-site trial showed that the intervention was successful in voluntarily recruiting and retaining participants regardless of delivery condition (i.e., ME vs. didactic) (20) and engaging Latinx participants in positive group processes in the motivational enhancement condition (21). These prior studies demonstrated that the adaptations of Project Options to each site facilitated engagement of Latinx youth thereby lowering barriers to participation and showed that mechanisms of change at the group level were favorable for Latinx participants. Findings from this study extend these promising results by examining the tentative utility of this EBI for changing drinking cognitions and outcomes for this underserved group across immigrant generation and gender. Findings also contribute to the field's discussion of when and whether to adapt EBIs to maximize benefits for underserved youth (12, 77). Additionally, it adds to the small, but growing body of innovative research implementing hybrid efficacy-effectiveness trials [e.g., (72)] in order to serve the community in the real-world given the high need.

### Implications

Together these results tentatively demonstrate that adapting an EBI to the local context and the developmental stage of adolescent participants might be an effective way to ensure its usefulness for Latinx youth of different immigrant generations. Although the intervention content was not specifically adapted to the culture of Latinx youth, the EBI's adaptation to the local context of each site might have captured aspects of the general cultural school environment in which these youth are developing, thereby meeting their needs. These adaptations are consistent with one of the data-driven arms proposed by Lau (12) wherein barriers to EBI engagement for underserved ethnocultural groups are eliminated by decreasing participation barriers while maintaining the style and content of the EBI. Therefore, adapting to the immediate context of youth might be particularly useful for school-based interventions that service the setting to maximize reach regardless of its continuously changing ethnic composition. As such, Project Options might be a promising intervention for Latinx youth.

### Limitations

Study findings must be considered within its limitations. Ethnic background and drinking experience were not considered in the initial randomization of participants to the ME or didactic conditions. As a result, not enough Latinx participants in each lifetime drinking experience category were present at each site to allow for nesting of observations by site or school. In addition, this led to differences in participant drinking profiles. For example, those randomized to the ME condition reported a higher average number of drinks per occasion and less positive cessation expectancies than those in the didactic style condition. As expected, the majority of Latinx participants in the BI across the three sites (Miami, Minneapolis, and Portland) were from Miami. While we attempted to account for these differences analytically, these findings might reflect the Latinx culture of Miami and may not be generalizable to other Latinx local contexts. Relatedly, we were unable to examine potential variation by sub-groups of Latinx youth; some studies have found differences in alcohol use by generation and Latinx sub-group [e.g., (25, 42, 78)]. Furthermore, since this multi-site clinical trial was not developed for Latinx youth specifically, important characteristics such as acculturation, ethnic identity, and familism were not assessed [see (79) for a review].

## Conclusions and Future Directions

In combination with prior evaluations of engagement/retention (20) and therapeutic processes (21) of this multi-site hybrid efficacy-effectiveness clinical trial of Project Options, this secondary analysis cautiously suggests that this voluntary EBI might be a promising intervention for Latinx youth of different immigrant generations. Findings tentatively suggest that adaptations to the local school context may capture cultural aspects important to the ecology of each site thereby also including key aspects of Latinx youth culture within each setting. Importantly, conducting an effectiveness study comparing this EBI to a control condition that appropriately mirrors treatment as usual will help elucidate whether Project Options content delivered through motivational enhancement style is effective for Latinx youth. In addition, results indicate that future research must examine whether and how EBIs serve the needs of Latinx youth by immigrant generation and gender. Additionally, examining whether attending sessions on specific topics is associated with intervention outcomes may also shed light on important intervention targets for Latinx youth. Only by addressing the mechanisms that lead to the differences in risk and protective factors by gender and immigrant generation will interventions successfully help curtail the health disparities encountered by Latinx youth as they transition to adulthood.

## Data Availability Statement

The data analyzed in this study is subject to the following licenses/restrictions: The datasets for this article are not publicly available because the study involved underage participants with alcohol use experience. We are not able to release data as part of the publication given the sensitivity of the data and our agreement with the institutions' Institutional Review Boards. Requests to access these datasets should be directed to Mark G. Myers, mgmyers@health.ucsd.edu.

## Ethics Statement

The studies involving human participants were reviewed and approved by the Institutional Review Boards at Florida International University, Reed College, University of Minnesota, and the University of California, San Diego. Written informed consent to participate in this study was provided by the participants' legal guardian/next of kin.

## Author Contributions

GB reviewed the literature, conducted statistical analyses, wrote initial manuscript drafts, and was responsible for edits. TG conducted the literature review, consulted on statistical analyses, and contributed to the writing and editing of the manuscript. KA was a co-investigator on the clinical trial, consulted on hypotheses, guided statistical analyses, and contributed to manuscript revisions. SB was a co-principal investigator on the clinical trial, provided feedback on drafts of the manuscript, and supervised the completion of the study. MM was a co-principal investigator on the clinical trial, consulted on statistical analyses, revised multiple drafts of the manuscript, and oversaw the completion of the study. All authors contributed to the article and approved the submitted version.

## Conflict of Interest

The authors declare that the research was conducted in the absence of any commercial or financial relationships that could be construed as a potential conflict of interest.

## Footnotes

^1^We use “Latinx” as a gender inclusive term that encompasses gender binary and non-binary individuals. We use “Latino” for findings specific to boys/men and “Latina” for findings specific to girls/women.
